# What Is Behavioral Complexity? Lay Perceptions of Characteristics of Complex Behavior

**DOI:** 10.3390/bs14080730

**Published:** 2024-08-22

**Authors:** Indita Dorina, Barbara Mullan, Mark Boyes, Thomas McAlpine

**Affiliations:** 1 School of Population Health, Faculty of Health Sciences, Curtin University, Perth 6102, Australia; indita.dorina@curtin.edu.au (I.D.); mark.boyes@curtin.edu.au (M.B.); thomas.mcalpine@curtin.edu.au (T.M.); 2 Behavioural Science and Health Research Group, enAble Research Institute, Faculty of Health Sciences, Curtin University, Perth 6102, Australia

**Keywords:** health behavior, environmental behavior, behavior change, habit, behavioral complexity

## Abstract

A behavior’s complexity may impact habit formation, with implications for habit-based public health and environmental intervention designs. However, there are varying conceptualizations of behavioral complexity, hindering the synthesis of findings. To develop a unified definition, the aim of this study was to explore perceptions of behavioral complexity and identify behaviors that exemplify aspects of complexity. Participants (*N* = 225) completed a questionnaire concerning the complexity of various health and environmental behaviors, the importance of complexity characteristics previously identified by researchers (novelty, difficulty, steps, planning, immediacy of reward, time, attention, skill, mental resources, self-efficacy, motivation for a behavior, and supportiveness of the context) and demographics. Participants considered all proposed characteristics to be important. Complex behaviors (e.g., abstaining from smoking and taking insulin shots), compared to simple behaviors (e.g., eating fruit and stretching), are more likely to be true to the previously identified characteristics. Perceived complexity is influenced by several salient characteristics. Results may contribute to a synthesized definition and underpin future research to better identify behavior change techniques to foster habitual behaviors of varying complexity. Hence, researchers, practitioners, and policymakers may identify common barriers and facilitators of behavior to target in interventions. However, further research is required to contextualize the findings.

## 1. Introduction

The majority of behavior change interventions are successful in changing behavior in the short term, but they often fail to result in the maintenance of newly adopted behaviors over time [1]. To design effective interventions, the active ingredients of interventions, or behavior change techniques, must be identified [2]. However, as behavior change techniques were developed to target specific mechanisms of action in behaviors [3], understanding the salient characteristics of behavior may be important to facilitate the identification of common mechanisms of action and, therefore, the relevant behavior change techniques.

Researchers have attempted to categorize behaviors in various ways to understand the underlying mechanisms of action. For example, meta-analyses may choose to categorize behaviors by functionality (e.g., health promotive, health risk, and illness detecting behaviors [4]) or further sub-divide categories into descriptive groups (e.g., risk, detection, physical activity, dietary, safer sex, and abstinence [5]). However, human behavior is complex, and to sufficiently understand the drivers of behavior to effectively change them, the complexity of behaviors needs to be considered to design effective interventions [6].

One of the most commonly noted consequences of behavioral complexity in behavior change research is that some behaviors are more complex to habitually implement or maintain than others [7]. This is important to consider when habits can encourage behaviors in a more automatic, rapid, and unconscious manner [8]. Therefore, due to habits being the default responses in specific contexts, they may trigger behaviors automatically, with minimal drain on self-regulatory resources and during lapses in motivation or situations with competing demands [7]. Hence, habits can facilitate behavioral engagement and maintenance with greater consistency [1] and understanding the characteristics of behavioral complexity may help interventions foster habitual behavior to improve its effectiveness.

Although researchers agree that some behaviors are more complex than others (e.g., active commuting involves multiple sub-actions and is more complex than taking a pill), the proposed characteristics that define behavioral complexity are subject to debate. Phillips and Mullan [7] argue that complexity is an inherent characteristic of behaviors themselves, involving the number of meaningful, separable (e.g., in instigation and execution) and substitutable sub-actions or steps, which may require greater time to prepare and execute intrinsic motivation to form and maintain habits. Contrastingly, Rebar, Rhodes [9] argue that rewards and behavioral complexity are related but distinct constructs, and complexity is not solely a characteristic of the behavior itself but an interaction between the behavior, context (e.g., barriers and facilitators), and actor (e.g., skill level, knowledge acquisition, and cognitive load). Similarly, Kaushal and Rhodes [10] consider behavioral complexity as the perceived difficulty of a behavior, influenced by an individual’s self-efficacy, with McCloskey and Johnson [11] agreeing but also considering the time, attention, and planning required to prepare and execute a behavior. Although McCloskey and Johnson [11] have identified their proposals of behavioral complexity characteristics via data-driven methods, other proposals have not yet been tested, and no studies to date have tested all proposals together to synthesize the findings.

With varying and debated conceptualizations of behavioral complexity within the field, research is needed to assess lay individual’s opinions of the importance of the proposed characteristics of behavioral complexity. Data-driven approaches are also needed to investigate the validity of the proposed characteristics. Then, a synthesized and evidence-based definition can be developed to inform the methods required for tailored public health and environmental interventions. Understanding the salient characteristics of behavior can inform researchers, practitioners, individuals, and policy-makers of the common barriers and facilitators of various behavior types to target when aiming to change behavior. The findings can inform the suitability of tailored intervention methods and education programs to change behavior.

### The Current Study

To work towards a unified definition of behavioral complexity, the aim of this study was to explore people’s perceptions of the characteristics of complexity previously proposed by researchers. A secondary aim was to identify typical behaviors that exemplify the varying degrees of complexity across the characteristics of behavioral complexity. Due to the exploratory nature of this study, there were no specific hypotheses.

## 2. Materials and Methods

### 2.1. Participants

Participants were recruited from February to June 2023 via a university undergraduate participant pool. Participants received course credit in return for completing the study. Two hundred and fifty-two participants completed the study, with 27 cases removed due to not providing consent, being under 18 years of age, missing over 50% of data, or being a duplicate response (e.g., determined by their provided identifiers). Two hundred and twenty-five participants were retained in the final sample.

Participants were aged between 18 and 51 years (*M* = 23.10, SD = 7.14). Most identified as women (75.1%), 20.4% as men, 2.7% as non-binary, and 1.8% did not indicate their gender. Most resided in Western Australia (95.6%), with the remaining 4.4% residing in Victoria, Queensland, and New South Wales, or did not indicate their state of residence. Most received a high school education (69.5%), 20.4% received a university degree or above, 8.4% received a TAFE certificate (vocational education training), and 1.8% did not indicate.

### 2.2. Measures

For complexity ratings of behaviors, the participants were asked to indicate if 24 behaviors were simple or complex for the average person to do on a regular basis (1 = simple, 2 = complex). Specifically, they were instructed, “Please indicate whether the following behaviors are simple or complex for the average person to do on a regular basis.” Behaviors were chosen based on previous meta-analyses of behavior change theories, which categorized behaviors by type, e.g., [5,12]. Upon participants indicating that a behavior was complex, they were then probed to indicate how complex they think that behavior is for the average person to do on a regular basis on a 7-point Likert scale (1 = not at all complex, 7 = extremely complex). Specifically, they were instructed, “Please indicate how complex (rather than simple) that behavior is for the average person to do on a regular basis.”

For factors of behavioral complexity, the participants were asked to indicate whether 12 factors of behavioral complexity, as proposed by the literature [7,9,10,11], were influential or not influential to how complex a behavior is deemed to be (1 = not influential, 2 = influential). Specifically, they were instructed, “Please indicate whether the following factors are influential to how complex a behavior is for the average person to do on a regular basis.” Proposed factors of behavioral complexity were the novelty, difficulty, number of steps, planning, the immediacy of reward, time and attention associated with a behavior, an individual’s skill level, mental resources, self-efficacy, and motivation, and the supportiveness of a behavior’s general context. Upon participants indicating a factor of behavioral complexity to be influential, they were then probed to indicate how influential they thought that factor was on a 7-point Likert scale (1 = not at all influential, 7 = extremely influential). Specifically, they were instructed, “Please rate that factor’s level of influence to how complex a behavior is for the average person to do a behavior on a regular basis.”

### 2.3. Procedure

Participants completed a 30-minute online survey on Qualtrics by clicking on a link within study advertisements. Participants were first displayed a participant information sheet and asked to provide consent by clicking a box to continue. Only after providing informed consent were participants permitted to continue with the survey. At the end of the survey, participants were thanked for their time and requested to enter their contact details. The University Human Research Ethics Committee approved this study.

### 2.4. Data Analysis

SPSS Version 28 [13] was used for data cleaning (e.g., case deletion for high proportions of missing data, missing values analysis, renaming variable labels, and assumptions testing) and assessing for frequency counts (e.g., proportions) and descriptives statistics of participant demographics and variables (e.g., range, means, and standard deviations). To integrate findings and identify behaviors that exemplify the characteristics of behavioral complexity, post-hoc analyses were conducted by two members of the research team. Researchers used Microsoft Excel to plot participants’ mean ratings of each behavior’s complexity into individual figures, each representing a characteristic of behavioral complexity. Based on the spread of normality of ratings, behaviors low in complexity were those rated < 4, and those high in complexity were rated > 4. Behaviors were then categorized as true (1) or untrue (−1) to each characteristic based on researchers’ knowledge of the scientific literature surrounding each behavior and characteristic of complexity. The final figures consisted of four quadrants, which represented four categories of behavior: (1) low complexity behaviors that were untrue to characteristics, (2) low complexity behaviors that were true to characteristics, (3) high complexity behaviors that were true to characteristics, and (4) high complexity behaviors that were untrue to characteristics. The frequency of which behaviors were plotted in each quadrant across all figures determined the best-fitting category for that behavior. Raincloud plots to visually display the spread of distributions in responses were produced using R Statistical Software Version 4.4.1 [14].

## 3. Results

### 3.1. Complexity Ratings of Behaviors

When participants were initially asked to rate behaviors as simple or complex, eating breakfast was least frequently rated as complex (10.7%). Contrastingly, applying sunscreen was most frequently rated as complex (73.3%). When participants were further probed on how complex each behavior was, which they only previously rated as complex, eating fruit was rated as the least complex (*M* = 2.97, SD = 1.06), while abstaining from smoking cigarettes was rated as the most complex (*M* = 5.05, SD = 1.56). See Table 1 for the complexity ratings for each behavior.

### 3.2. Ratings of Influence for the Proposed Factors of Behavioral Complexity

When participants were initially asked to rate the proposed factors of behavioral complexity as influential or not influential, the immediacy of rewards was least frequently rated as influential (74.7%). Contrastingly, the time associated with a behavior was most frequently rated as influential (92.4%). When participants were further probed on how influential factors were, which they only previously rated were influential, there was little variability between ratings, with the novelty of a behavior rated as the least influential (*M* = 4.68, SD = 1.34) and motivation rated as the most influential (*M* = 5.40, SD = 1.23). See Table 2 for the ratings of influence for the proposed factors of behavioral complexity.

### 3.3. Trends in Behavioral Complexity Categorizations

Across all figures that represented the four categories of behavior (See Supplementary Materials S1), behaviors were most frequently categorized as highly complex and true to characteristics (40.28%). This was followed by behaviors categorized as low complexity and untrue to characteristics (29.51%), behaviors categorized as low complexity and true to characteristics (24.65%), and behaviors categorized as high complexity and untrue to characteristics (5.56%). See Table 3 for the behaviors categorized across characteristics of behavioral complexity.

**Figure 1:**
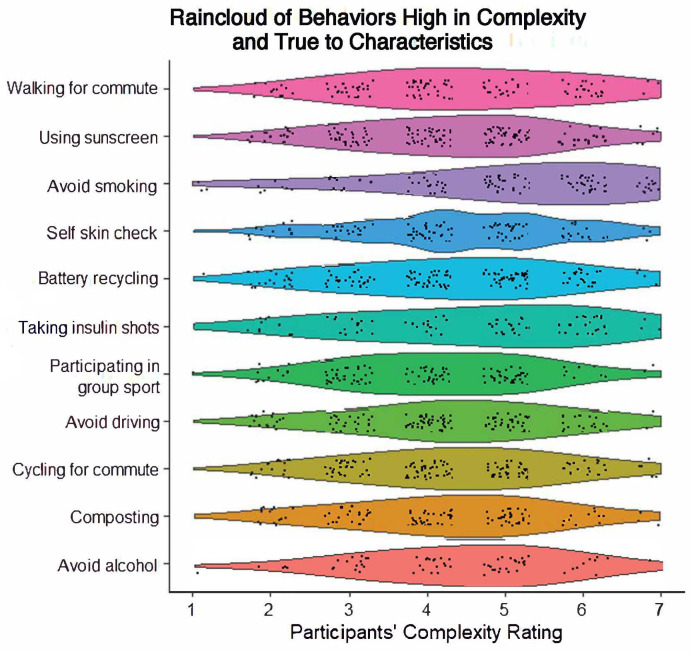
Raincloud plot of behaviors high in complexity and true to characteristics.

There was good consistency in behaviors, which were highly complex and true to characteristics. This category always included abstaining from smoking cigarettes (*M* = 5.05, 100% identified across all characteristics), taking insulin shots (*M* = 4.51, 100% identified), limiting alcohol consumption (*M* = 4.26, 100% identified), cycling for commute (*M* = 4.25, 100% identified), and composting (*M* = 4.14, 100% identified). Other commonly included behaviors were performing self-skin checks (*M* = 4.35, 91.67% identified), walking for commuting (*M* = 4.36, 83.33% identified), using sunscreen (*M* = 4.22, 83.33% identified), and partaking in group sports (*M* = 4.02, 83.33% identified). Behaviors in this category visually indicate a slightly right-skewed or right-skewed distribution. See Figure 1 for the behaviors in this category, with a visual display of the spread of distributions in the complexity ratings.

**Figure 2:**
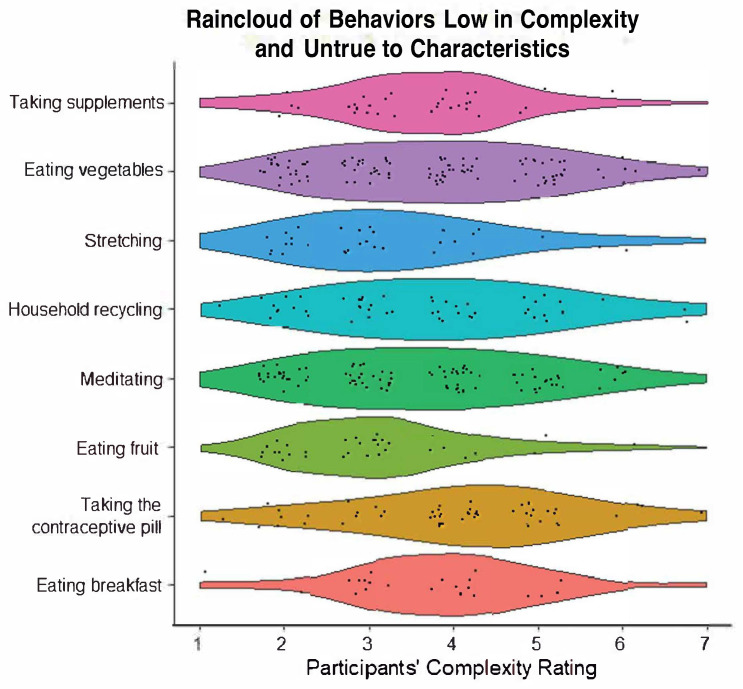
Raincloud plot of behaviors low in complexity and untrue to characteristics.

There was also good consistency in behaviors that were low in complexity and untrue to characteristics. This category always included eating fruits (*M* = 2.97, 100% identified) and stretching (*M* = 3.09, 100% identified). Other commonly included behaviors were general household recycling (*M* = 3.73, 91.61% identified), taking the contraceptive pill (*M* = 3.96, 91.61% identified), and taking vitamin supplements (*M* = 3.40, 83.33% identified). Most behaviors in this category visually indicate a left-skewed distribution. See Figure 2 for the behaviors in this category, with a visual display of the spread of distributions in the complexity ratings.

**Figure 3:**
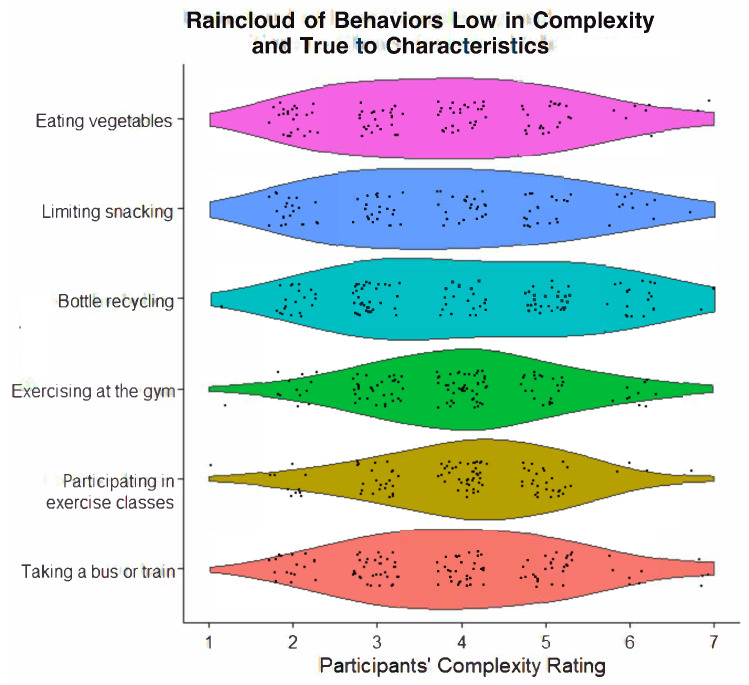
Raincloud plot of behaviors low in complexity and true to characteristics.

Similarly, there was also good consistency in behaviors that were low in complexity and true to characteristics. This category always included exercising at the gym (*M* = 3.86, 100% identified) and taking a bus or train for commuting (*M* = 3.89, 100% identified). Other commonly included behaviors were limiting snacking (*M* = 3.69, 91.67% identified) and partaking in exercise classes (*M* = 3.92, 91.67% identified). There was high heterogeneity in some behaviors in this category, visually indicating a slightly left-skewed or normal distribution. See Figure 3 for the behaviors in this category, with a visual display of the spread of distributions in the complexity ratings.

**Figure 4:**
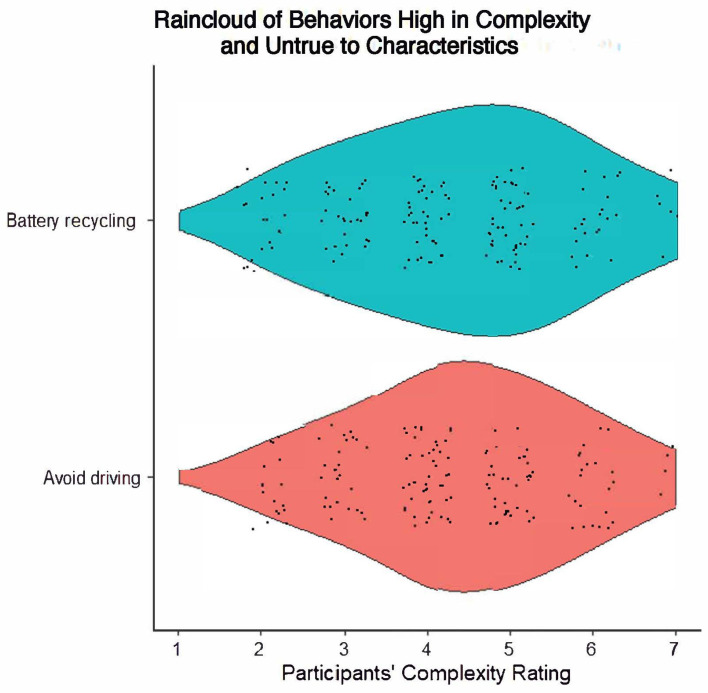
Raincloud plot of behaviors high in complexity and untrue to characteristics.

However, behaviors were rarely categorized as highly complex and untrue to characteristics. There was the least consistency within this category across factors, but this category included recycling batteries (*M* = 4.25, 41.67% identified) and avoiding driving for commuting (*M* = 4.25, 33.33% identified). Behaviors in this category visually indicate a slightly right-skewed distribution. See Figure 4 for the behaviors in this category, with a visual display of the spread of distributions in the complexity ratings.

## 4. Discussion

To work toward a unified definition, the aim of this study was to explore lay individuals’ perceptions of the characteristics of behavioral complexity that were previously proposed by researchers. A secondary aim was to identify typical behaviors that exemplify the varying degrees of complexity across these characteristics. Participants rated all characteristics to be influential to the complexity of behaviors, with little variability in the ratings of influence between characteristics. Typical behaviors that reflect the varying degrees of complexity across the characteristics of behavioral complexity were also identified. Highly complex behaviors, which were true to characteristics, include abstaining from smoking cigarettes, taking insulin shots, limiting alcohol consumption, cycling for commuting, and composting. Low complexity behaviors, which were untrue to characteristics, include eating fruit and stretching. Finally, low complexity behaviors, which were true to characteristics, were exercising at the gym and taking a bus or train for commuting. Few behaviors were highly complex and untrue to characteristics. Findings support the idea that complex behaviors, compared to simple behaviors, are more likely to be true to the characteristics.

### 4.1. Characteristics of Behavioral Complexity

Participants perceived all proposed characteristics as influential to the complexity of a behavior. Researchers have previously speculated that characteristics include the number of steps and motivation required for a behavior [7], supportiveness of the context, an individual’s skill, knowledge acquisition and cognitive load [9], perceived difficulty of a behavior, self-efficacy [10], and the time, attention, and planning involved in a behavior [11]. However, this study expands on previous research using data-driven methods to synthesize and test these ideas to reveal that all characteristics are influential. Although findings support all individually proposed perspectives of behavioral complexity, individual perspectives alone may not be sufficient to define them. Based on the current results, which found that complex behaviors are more likely to be true to characteristics than simple behaviors, we propose that a synthesized definition of behavioral complexity may consider the additive function of all proposed characteristics collectively. Specifically, although behaviors may not always be true to all characteristics (e.g., due to potential variability in individual and situational factors), behaviors possessing a greater number of characteristics at any given time may contribute to greater complexity. This notion aligns with complex systems approaches to behavior change, considering the interconnectedness of factors of behavior, heterogeneity across individuals, and non-linear dynamics [15]. From this perspective, complexity may be influenced by various levels of systems or scales [16] ranging from individuals and communities to broader policies. Future research may test the validity of this definition by assessing within and between-person fluctuations (e.g., via ecological momentary assessment methods) in individuals’ perceived complexity of the identified exemplar behaviors and how true each behavior is to each characteristic of complexity. This is of importance to improve theoretical understandings regarding the mechanisms of automatic factors of behavior. Some of the participant ratings were unexpected (e.g., physical activity behaviors as low complexity) in that they do not align with definitions of complexity used in previous research to establish the influence of behavioral complexity as a moderator of the habit–behavior relationship [17] and implicit attitude–behavior relationship [18,19].

Current findings indicate the importance of considering behavioral, individual, and contextual factors when aiming to tailor intervention methods to foster habit formation across behaviors of varying complexity. As behavior change techniques are designed to target specific mechanisms of action [3], relevant behavior change techniques may be identified and implemented in public health and environmental interventions aiming to change behaviors with certain characteristics. For example, one common characteristic of complex behaviors, such as abstaining from smoking, identified in this study, is that the behavior commonly occurs in an unsupportive context. Relevant behavior change techniques that can be implemented to foster supportive environmental contexts include restructuring the physical and social environment or encouraging social support [2]. Similarly, complex behaviors that require developed skills and strong self-efficacy, such as administering insulin shots, may be relevant to behavior change techniques such as problem-solving or behavioral practice and rehearsal [2]. Habit-based public health and environmental intervention methods may be tailored to non-changeable characteristics of complex behaviors, such as the environmental barriers or inherent characteristics of a behavior itself (e.g., by applying implementation intentions or action and coping planning strategies to prepare for and overcome difficult situations when intentions are likely to fail [20]). However, researchers may amend the changeable characteristics of habit formation in complex behaviors by fostering individuals’ intrinsic motivation, availability of mental resources, skill development, and self-efficacy while reducing their perceived difficulty of a behavior.

There was little variability in the ratings of influence between characteristics (*M* = 4.68 − 5.40, SD = 1.12 − 1.34), indicating the importance of all characteristics for most individuals, at least in certain situations. However, future research may use qualitative methodology to contextualize and gain further insight into when and how characteristics are most influential, including the prominence of characteristics depending on the individual or context. This is important to note due to the heterogeneity in the complexity ratings of behaviors visually displayed in raincloud plots. Understanding this heterogeneity may contribute to more nuanced and relevant intervention designs [21]. One area of research may be to explore how characteristics interact to influence habits and habitual behavior, as there are mixed proposals on how this may be influenced by behavioral complexity. For example, Rebar and Rhodes [9] proposed that the ease in behavioral engagement may depend on dynamic interactions between behavioral, individual, and contextual factors, while Gardner and Lally [22] propose that habitual processes for behaviors are equal regardless of complexity, and Phillips and Mullan [7] propose that complex behaviors require greater intrinsic motivation and take longer to become habitual, therefore producing differences in the outcomes of habit impulses and intervention techniques required [23]. Alternatively, other factors not considered in the current research, which may be more characteristic of behavioral complexity, should be explored, as the current research only assessed participants’ perceptions guided by researchers’ proposals. For example, previously proposed behavioral characteristics include the likelihood of a behavior’s consequence, severity, and tangibility of a behavior’s impact, and the emotionality and nature of a behavior (e.g., as approach or avoidance) [24].

### 4.2. Typical Behaviors of Complexity Characteristics

Behaviors were most often and consistently categorized as highly complex and true to characteristics (e.g., abstaining from smoking cigarettes, taking insulin shots, limiting alcohol consumption, cycling for commuting, and composting). There was also consistency in low-complexity behaviors that were untrue to characteristics (e.g., eating fruit and stretching). The identified typical behaviors may be implemented in future research for theory testing and research with the aim of optimizing habit-based interventions across behaviors of varying complexity. It is important to have consistency in the behaviors implemented in habit research to facilitate the synthesis of findings and the accumulation of knowledge required to inform a broader understanding of habit formation and disruption for long-term behavior change [25]. Additionally, only a few behaviors were categorized as highly complex and untrue to characteristics, with inconsistencies in behaviors included in this category. Therefore, findings are consistent with researchers’ conceptualizations, where complex behaviors, compared to simple behaviors, are more likely to possess or be influenced by these factors [7,9,10,11].

However, there was still good consistency in behaviors that were low in complexity and true to characteristics. Behaviors always included in this category were exercising at the gym and taking a bus or train for commuting. Findings are inconsistent with the literature as it is expected that behaviors that are characteristic or expected to be influenced by the proposed factors would be more complex [7,9,10,11]. However, frequently performed behaviors engaged in a more automatic manner are typically perceived as less complex [11]. Therefore, it is possible that these behaviors may not be perceived as complex, although still true to characteristics, as individuals may already engage in the behavior habitually or perceive others to. Perceived behavioral control (i.e., self-efficacy as an identified characteristic in this study [26]), may be related to the challenge level in non-habitually or frequently performed tasks [27] and should be strengthened to facilitate these behaviors (e.g., by providing the resources and opportunities required).

Alternatively, findings may be attributed to behaviors being categorized post-hoc by researchers as being true to the characteristics or not. Future research should investigate participants’ perceptions of how similar or influenced by characteristics these behaviors may be, as behaviors may only be somewhat similar or influenced despite still being similar or influenced to some degree.

### 4.3. Strengths and Limitations

This study was the first that we are aware of to explore lay individuals’ perceptions of the characteristics of behavioral complexity based on researchers’ proposals. Findings may help resolve the currently mixed proposals in the literature to work towards a synthesized definition of behavioral complexity. This definition may consider the additive function of the identified characteristics of behavioral complexity, whereby behaviors may not always be true to all characteristics, but greater complexity is contributed to by possessing a greater number of characteristics at any given time. Therefore, a framework may be developed to inform tailored habit-based interventions and assessment methods for habit across behaviors of varying complexity [7]. This framework can guide public health and environmental intervention designs, the methods implemented by practitioners to encourage individual behavior change, and future research aiming to optimize interventions by tailoring behavior change techniques to the behavior types of interest and the relevant characteristics of behavior identified in this study. However, it should be noted that further research is required to test this definition before applying it in future research. For example, future research may explore ways of objectively assessing behavioral complexity to counteract self-perceptions, as it is known that individuals who frequently perform behaviors may perceive them as less complex [11].

Additionally, this study has identified typical behaviors that reflect the varying types and degrees of complexity across the proposed characteristics. The identified behaviors can be employed in future research to assess the influence of behavioral complexity in habitual processes more consistently. Therefore, findings from this research may help address one priority raised by expert panels in the behavior change field to further theory and evidence for intervention development by understanding the influence of behavioral complexity on habits [25]. Similarly, the identified behaviors can be used to assess the utility of theoretical frameworks to behaviors of varying complexity, as there is evidence that the importance of constructs in behavioral models, such as temporal self-regulation theory, may differ depending on a behavior’s complexity [12].

However, categorizations were conducted post-hoc by researchers to determine if each behavior was similar or expected to be influenced by the characteristics of behavioral complexity. Therefore, it is not known if or to what extent lay individuals perceive these behaviors to be true to the characteristics. Future research is required to confirm how similar behaviors are and how influenced each behavior is by the characteristics so as to determine, more robustly, the typical examples of behaviors of varying complexity. One approach may be to apply a latent profile analysis or similar methods to identify subgroups of behaviors based on the similarity of latent characteristics [28], as the current study was limited due to the structure of the data and could not apply the methods.

Additionally, the population average for the complexity of behaviors and the importance of the characteristics to behavioral complexity may be difficult to interpret due to the questioning style, where participants were first asked to choose a category via a binary response option and then prompted by a Likert scale format question to avoid central tendency bias [29]. It is commonly observed that binary response options are equally reliable and valid to multi-category formats, but multi-choice formats may be more suitable to assess individuals’ attitudes [30]. Although a mid-point in the follow-up question was included in this study to allow participants to reassess their response, future research may replicate the current study to more robustly assess the true average of behavioral complexity ratings and the importance of complexity characteristics.

Further, only the importance of existing conceptualizations in the literature was assessed so that other possible characteristics of behavioral complexity may not be captured in the current study. Future research may use qualitative methodology to identify any influential factors not considered. However, the current study has included the most pertinent proposals of behavioral complexity in the literature, presented from extensive evidence surrounding habit formation across various behavior types. Additionally, further contextualization of findings or moderators of behavioral complexity may be explored as the included behaviors in the current study are not comparable based on frequency, duration, and context. Differences in the propositions for each behavior need to be considered, as for behaviors to effectively achieve the desired outcome, they must follow the recommended guidelines [31].

## 5. Conclusions

To work towards a unified definition, the aim of this study was to explore lay individuals’ perceptions of the characteristics of behavioral complexity previously proposed by researchers. A secondary aim was to identify typical behaviors that exemplify the varying degrees of complexity across the factors of behavioral complexity. Participants rated all factors to be influential to the complexity of behaviors but with little variability in the ratings between factors. Typical behaviors that reflect the varying degrees of complexity across all proposed factors were also identified and may be consistently implemented in future research to foster the synthesis of findings. Findings have implications for the development of a synthesized definition of behavioral complexity. Therefore, future research can better identify the required techniques to measure and foster habit formation and maintenance across behaviors of varying complexity. Researchers, practitioners, and policy-makers may also identify the common barriers and facilitators of various behavior types to target in public health and environmental interventions.

## Figures and Tables

**Table 1:** Mean complexity ratings of behaviors.

Behavior	Proportion of Responses Indicating Complex % (*n* = 225)	Degree of Complexity of Behaviors Rated Complex
		*M* (SD)
Abstaining from smoking entirely	56.9	5.05 (1.56)
Taking insulin shots as recommended and appropriately dosed by doctors at least once a day	40.4	4.51 (1.51)
Walking for commute instead of driving, whenever possible	54.2	4.36 (1.41)
Doing self-skin checks on all parts of the body once every three months	50.2	4.35 (1.34)
Limiting alcohol consumption to no more than 10 standard drinks a week and no more than 4 standard drinks on any one-day	30.2	4.26 (1.23)
Recycling all batteries at designated battery recycling facilities as needed	72.4	4.25 (1.42)
Cycling for commute instead of driving, whenever possible	68.0	4.25 (1.31)
Abstaining from driving a car for commute, whenever possible	70.2	4.25 (1.32)
Using one tablespoon of sunscreen on each and all exposed body parts 20 min before sun exposure and re-applying every two hours	73.3	4.22 (1.29)
Composting all compostable items as needed	51.6	4.14 (1.31)
Partaking in group sports activities (e.g., AFL, netball) for at least an hour at least once a week	53.3	4.02 (1.22)
Recycling all accepted bottles at bottle recycling depots as needed	56.4	3.98 (1.45)
Taking the contraceptive pill once a day, around the same time each day	24.4	3.96 (1.33)
Partaking in exercise classes (e.g., Pilates, yoga, dancing) for at least 30 min, at least once a week	48.4	3.92 (1.13)
Taking a bus or train for commute instead of driving, whenever possible	54.2	3.89 (1.27)
Exercising at the gym for at least 30 min a few times a week	57.3	3.86 (1.15)
Eating one breakfast meal per day	10.7	3.75 (1.19)
Recycling all general plastics, paper, or cardboard in a general household recycling bin as needed	23.1	3.73 (1.40)
Eating five servings of vegetables per day	47.6	3.70 (1.35)
Eating no more than one serving of unhealthy snacks per day	44.4	3.69 (1.38)
Meditating for at least 10 min at least once a day	45.8	3.52 (1.36)
Taking vitamin supplements as instructed, at least once a day	15.6	3.40 (1.09)
Stretching for at least 5 min a few times a week	14.2	3.09 (1.17)
Eating two servings of fruits per day	15.1	2.97 (1.06)

Note. *n* between the proportion of responses indicating behaviors as complex and the degree of complexity of behaviors that were rated complex may vary, as participants were only prompted if first indicating that a behavior was complex rather than simple.

**Table 2:** Mean ratings of influence for the proposed factors of behavioral complexity.

Proposed Factor of Behavioral Complexity	Proportion of Responses Indicating Influential % (*n* = 223)	Degree of Influence of Factors Rated Influential
		*M* (SD)
Motivation to do the behavior	92.0	5.40 (1.23)
The behavior is difficult to do	91.6	5.39 (1.14)
The behavior requires time for preparation and doing	92.4	5.21 (1.17)
The behavior requires multiple steps to complete	90.7	5.15 (1.23)
The behavior requires planning	92.0	5.05 (1.12)
Skill level to do the behavior	86.7	4.92 (1.16)
Beliefs around being able to successfully do the behavior	88.4	4.90 (1.30)
The amount of mental resources available	91.6	4.90 (1.22)
The behavior requires focused attention	91.6	4.48 (1.22)
Contexts that can hinder or encourage the behavior	91.1	4.83 (1.21)
The reward of the behavior is not experienced until time has passed since initially doing it	74.7	4.74 (1.34)
The behavior is novel or new	84.9	4.68 (1.34)

Note. *n* between the proportion of responses indicating factors as influential and the degree of influence of factors that were rated influential may vary, as participants were only prompted if first indicating that a factor was influential rather than not influential.

**Table 3:** Categorizations of behaviors across the proposed factors of behavioral complexity.

Behavior	Individual Motivation	Behavior Is Difficult	Behavior Requires Time	Behavior Has Multiple Steps	Behavior Requires Planning	Individual Skill Level	Individual Beliefs of Success	Individual Mental Resources	Behavior Requires Attention	Behavior Is Hindered by Context	Behavior Has Distal Rewards	Behavior Is Novel
Avoid smoking	+	+	+	+	+	+	+	+	+	+	+	+
Taking insulin shots	+	+	+	+	+	+	+	+	+	+	+	+
Walking for commute	+	+	+	+	+	−	+	+	−	+	+	+
Doing self-skin checks	+	+	−	+	+	+	+	+	+	+	+	+
Limiting alcohol	+	+	+	+	+	+	+	+	+	+	+	+
Battery recycling	+	−	+	+	+	−	+	−	−	+	+	+
Cycling for commute	+	+	+	+	+	+	+	+	+	+	+	+
Avoid driving	+	+	+	−	+	−	+	−	−	+	+	+
Using sunscreen	+	−	+	+	+	−	+	+	+	+	+	+
Composting	+	+	+	+	+	+	+	+	+	+	+	+
Partaking in group sport	+	+	+	+	+	+	+	+	+	−	−	+
Bottle recycling	+	−	+	+	+	−	−	−	−	+	+	+
Taking the contraceptive pill	−	−	−	−	+	−	−	−	−	−	+	−
Partaking in exercise classes	+	+	+	+	+	+	+	+	+	−	+	+
Taking a bus or train	+	+	+	+	+	+	+	+	+	+	+	+
Exercising at the gym	+	+	+	+	+	+	+	+	+	+	+	+
Eating breakfast	+	−	−	+	+	−	−	−	−	+	+	−
Household recycling	−	−	−	−	−	−	−	−	−	−	+	−
Eating vegetables	−	−	−	−	+	−	+	−	−	+	+	+
Limiting snacking	−	+	+	−	+	+	+	+	+	+	+	+
Meditating	−	−	−	−	−	−	−	−	+	−	+	+
Taking supplements	−	−	−	−	−	−	−	−	−	−	+	+
Stretching	−	−	−	−	−	−	−	−	−	−	−	−
Eating fruits	−	−	−	−	−	−	−	−	−	−	−	−

Note. + = Behavior is similar or expected to be highly influenced by that factor. − = Behavior is not similar or not expected to be highly influenced by that factor.

## Data Availability

The data described in this article are openly available in the Open Science Framework at https://osf.io/z8w93/ (accessed on 19 August 2024).

## Supplementary Materials

The following supporting information can be downloaded at https://www.mdpi.com/article/10.3390/bs14080730/s1. Plots 1–12 indicate the categories of behaviors across each proposed factor of behavioral complexity.
