# Supplementary material for: What Is Behavioral Complexity? Lay Perceptions of Characteristics of Complex Behavior
Source: Behav Sci (Basel). 2024 Aug 22;14(8):730. doi: 10.3390/bs14080730 (PMC11351980; doi:10.3390/bs14080730)
Supplement: Supplementary file 1 [file behavsci-14-00730-s001.zip › behavsci-3102248-supplementary.pdf]

Supplementary Material S1

Plots 1-12 indicating the categories of behaviors across each proposed factor of behavioral complexity.

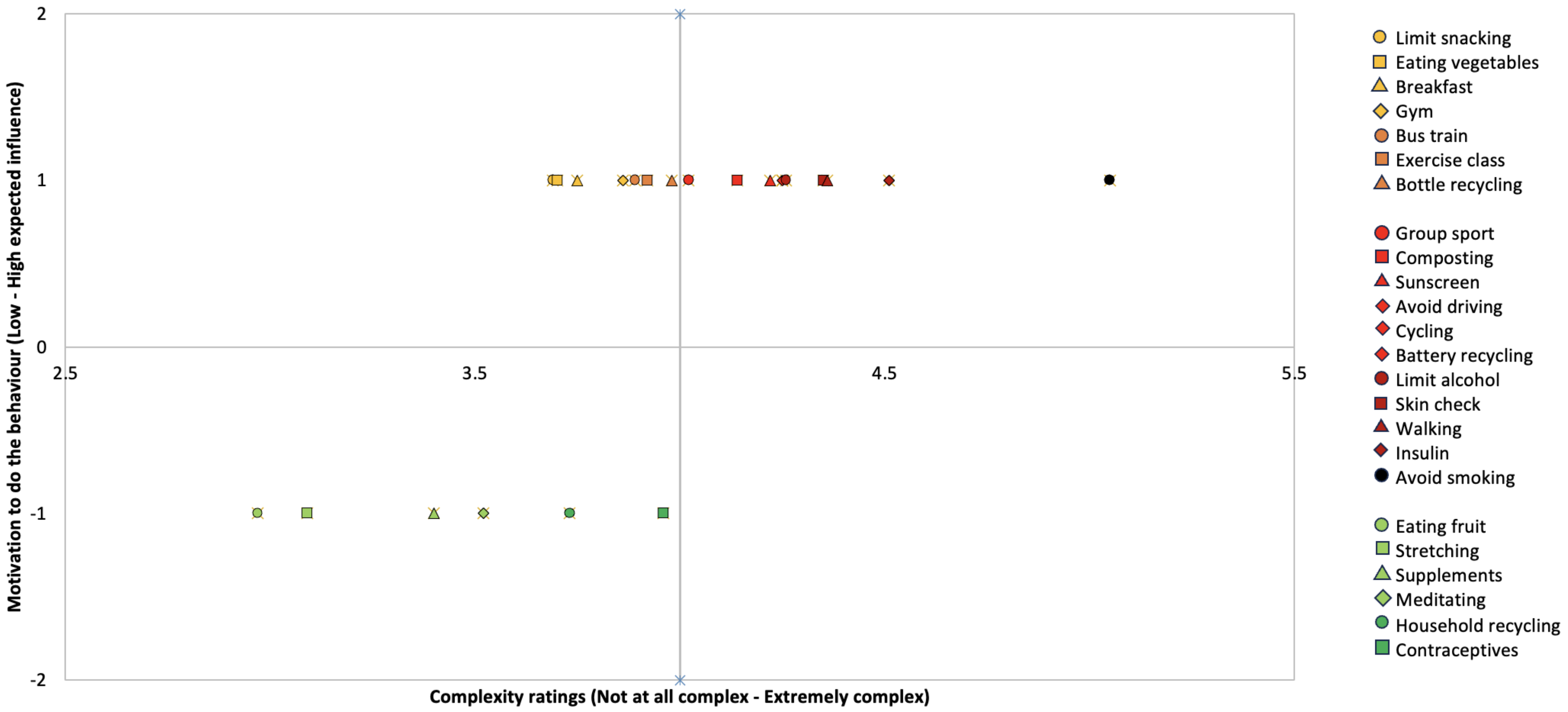

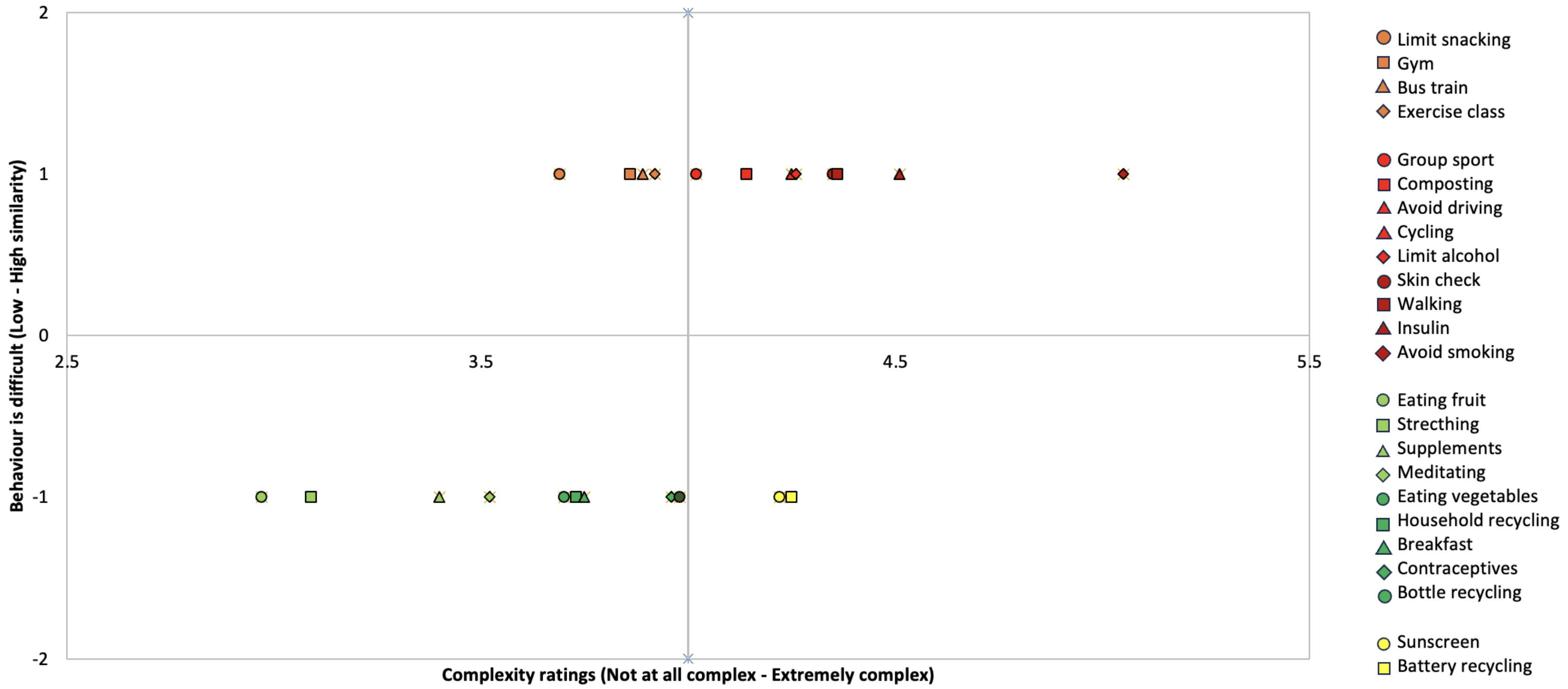

Behaviour is difficult (Low - High similarity)
Complexity ratings (Not at all complex - Extremely complex)
2
1
0
-1
-2
2.5
3.5
4.5
5.5
Limit snacking
Gym
Bus train
Exercise class
Group sport
Composting
Avoid driving
Cycling
Limit alcohol
Skin check
Walking
Insulin
Avoid smoking
Eating fruit
Strecthing
Supplements
Meditating
Eating vegetables
Household recycling
Breakfast
Contraceptives
Bottle recycling
Sunscreen
Battery recycling

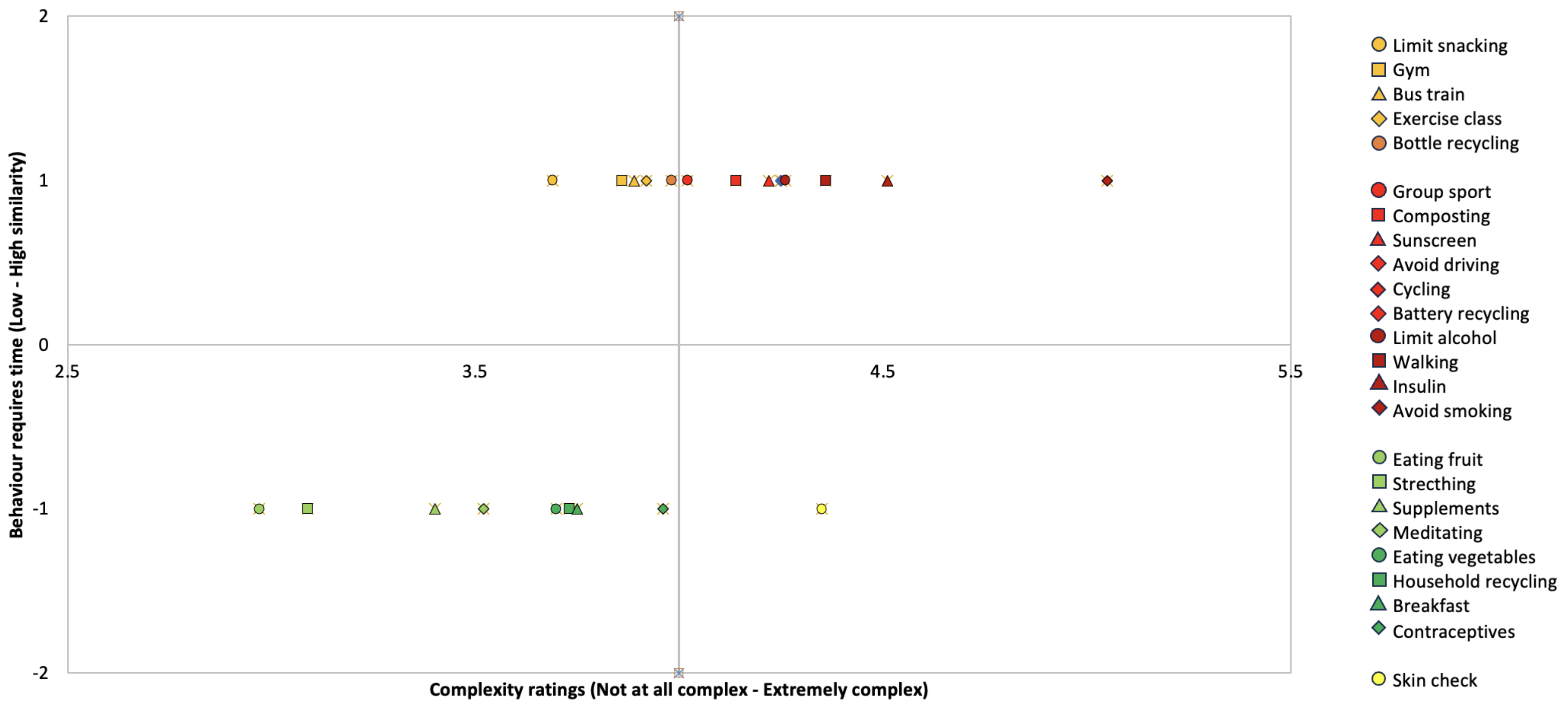
2
1
0
-1
-2
2.5
3.5
4.5
5.5
Behaviour requires time (Low - High similarity)
Complexity ratings (Not at all complex - Extremely complex)
Limit snacking
Gym
Bus train
Exercise class
Bottle recycling
Group sport
Composting
Sunscreen
Avoid driving
Cycling
Battery recycling
Limit alcohol
Walking
Insulin
Avoid smoking
Eating fruit
Strecthing
Supplements
Meditating
Eating vegetables
Household recycling
Breakfast
Contraceptives
Skin check

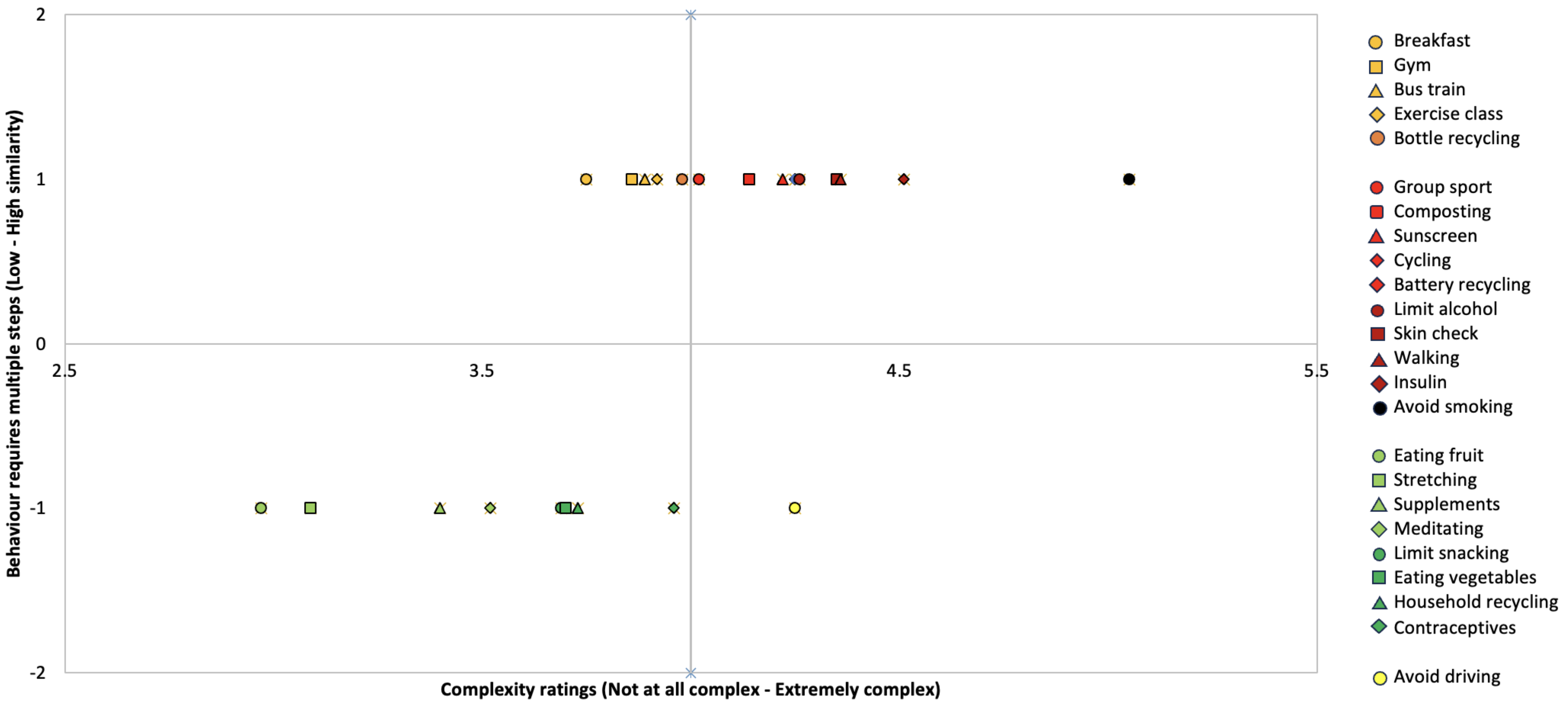
2
1
0
-1
-2
2.5
3.5
4.5
5.5
Behaviour requires multiple steps (Low - High similarity)
Complexity ratings (Not at all complex - Extremely complex)
Breakfast
Gym
Bus train
Exercise class
Bottle recycling
Group sport
Composting
Sunscreen
Cycling
Battery recycling
Limit alcohol
Skin check
Walking
Insulin
Avoid smoking
Eating fruit
Stretching
Supplements
Meditating
Limit snacking
Eating vegetables
Household recycling
Contraceptives
Avoid driving

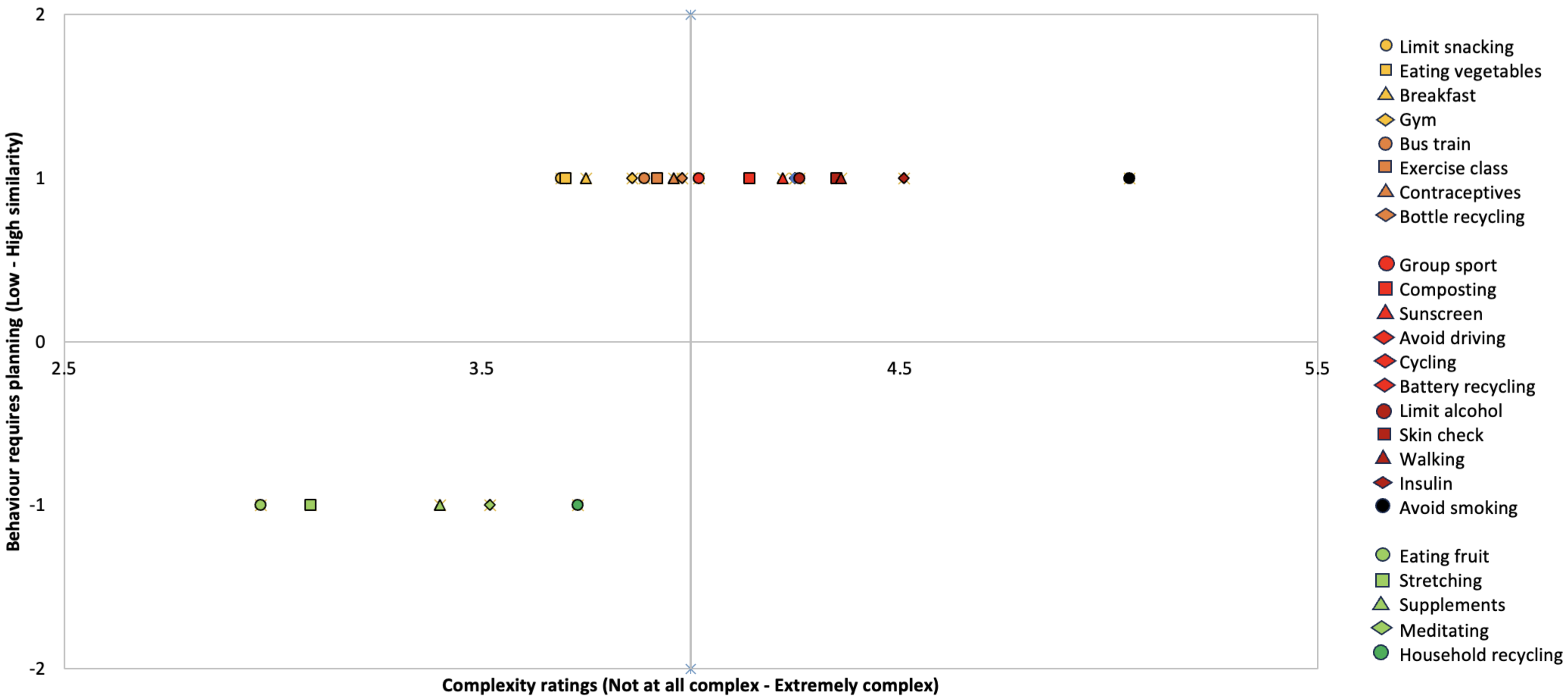
2
1
0
-1
-2
2.5
3.5
4.5
5.5
Behaviour requires planning (Low - High similarity)
Complexity ratings (Not at all complex - Extremely complex)
Limit snacking
Eating vegetables
Breakfast
Gym
Bus train
Exercise class
Contraceptives
Bottle recycling
Group sport
Composting
Sunscreen
Avoid driving
Cycling
Battery recycling
Limit alcohol
Skin check
Walking
Insulin
Avoid smoking
Eating fruit
Stretching
Supplements
Meditating
Household recycling

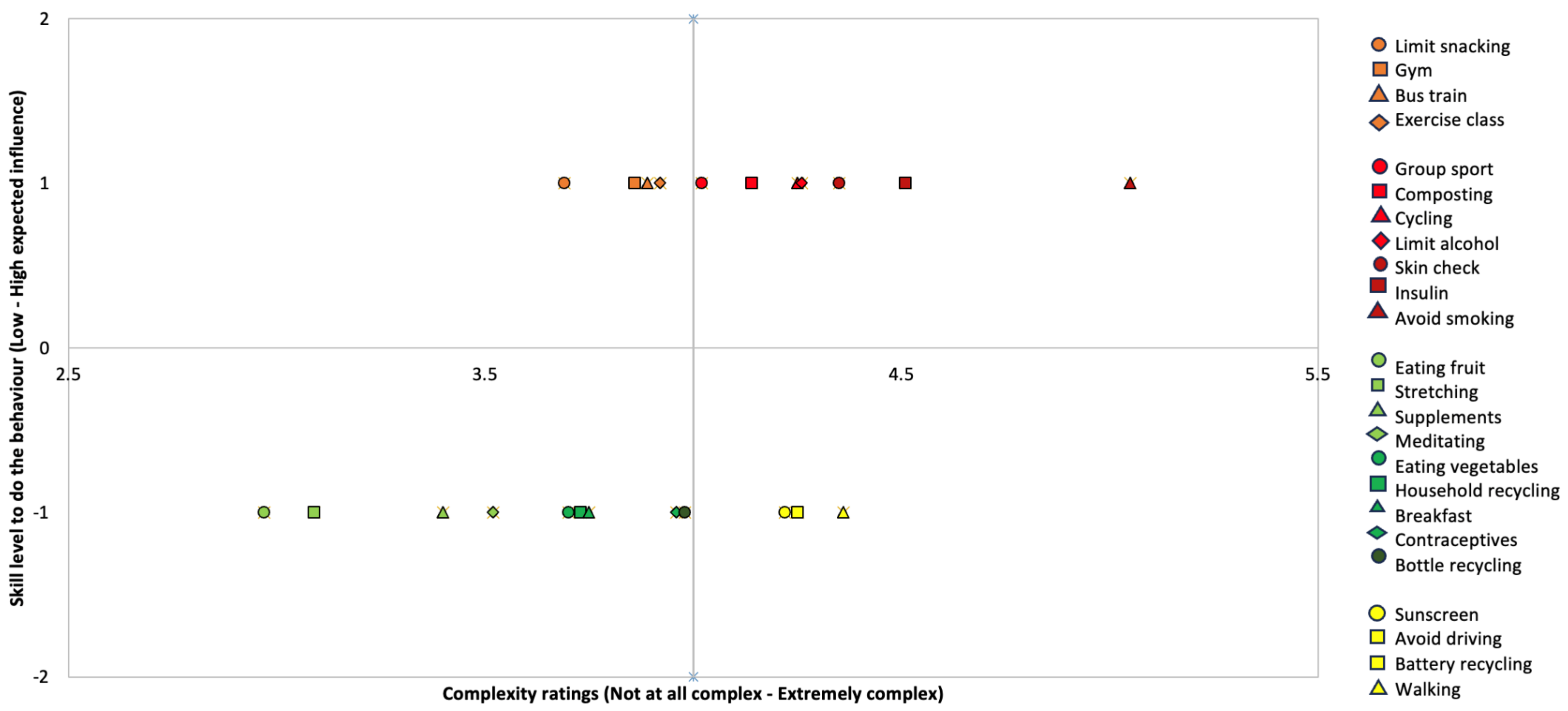
2
1
0
-1
-2
2.5
3.5
4.5
5.5
Skill level to do the behaviour (Low - High expected influence)
Complexity ratings (Not at all complex - Extremely complex)
Limit snacking
Gym
Bus train
Exercise class
Group sport
Composting
Cycling
Limit alcohol
Skin check
Insulin
Avoid smoking
Eating fruit
Stretching
Supplements
Meditating
Eating vegetables
Household recycling
Breakfast
Contraceptives
Bottle recycling
Sunscreen
Avoid driving
Battery recycling
Walking

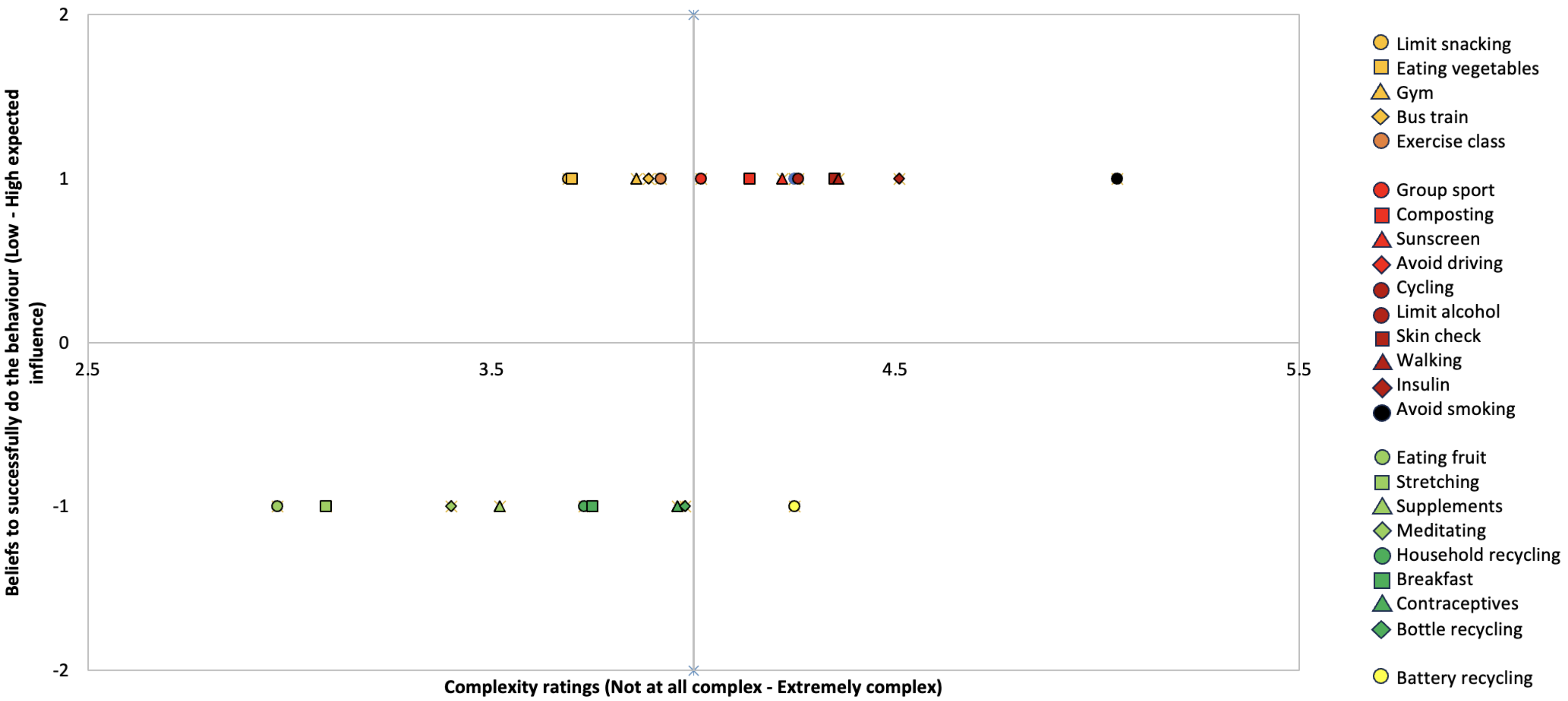
Beliefs to successfully do the behaviour (Low - High expected influence)
2
1
0
-1
-2
2.5
3.5
4.5
5.5
Complexity ratings (Not at all complex - Extremely complex)
Limit snacking
Eating vegetables
Gym
Bus train
Exercise class
Group sport
Composting
Sunscreen
Avoid driving
Cycling
Limit alcohol
Skin check
Walking
Insulin
Avoid smoking
Eating fruit
Stretching
Supplements
Meditating
Household recycling
Breakfast
Contraceptives
Bottle recycling
Battery recycling

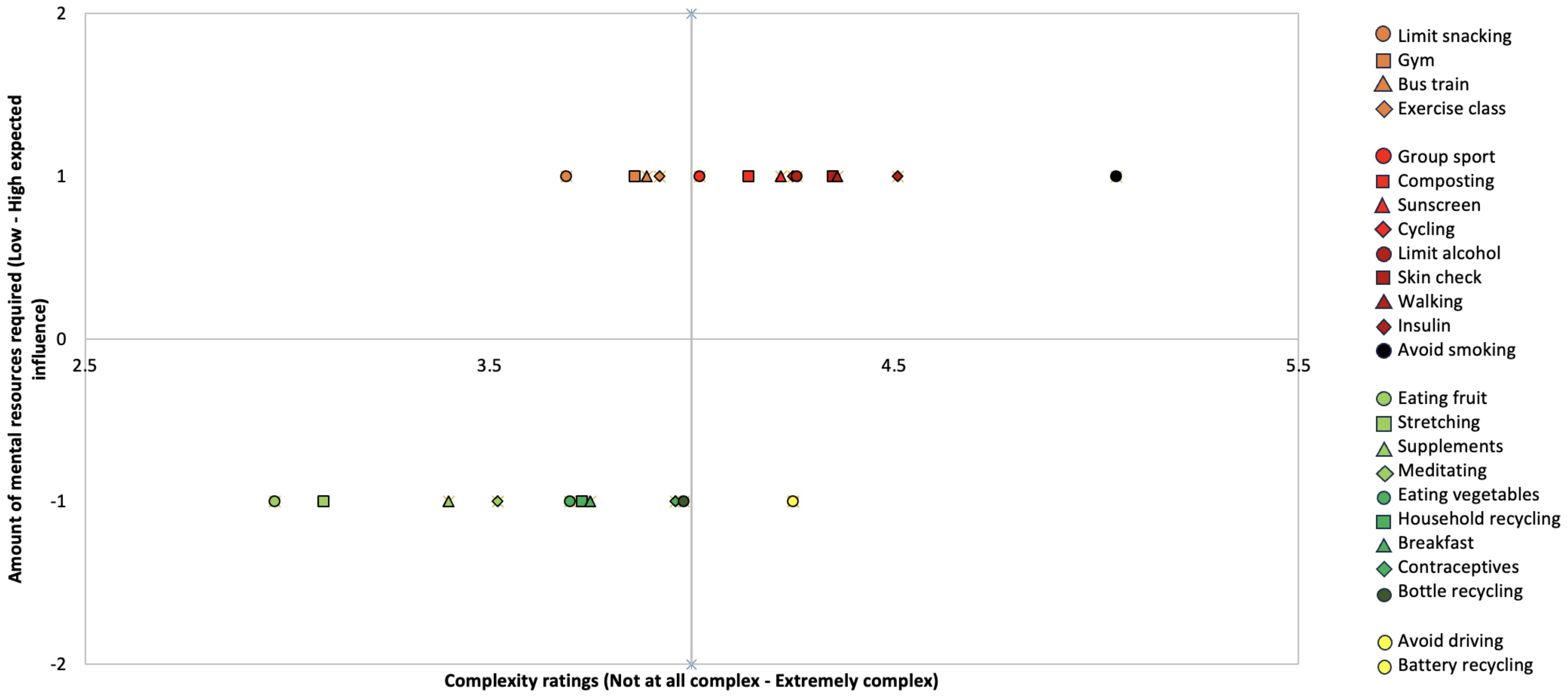
Amount of mental resources required (Low - High expected influence)
2
1
0
-1
-2
2.5
3.5
4.5
5.5
Complexity ratings (Not at all complex - Extremely complex)
Limit snacking
Gym
Bus train
Exercise class
Group sport
Composting
Sunscreen
Cycling
Limit alcohol
Skin check
Walking
Insulin
Avoid smoking
Eating fruit
Stretching
Supplements
Meditating
Eating vegetables
Household recycling
Breakfast
Contraceptives
Bottle recycling
Avoid driving
Battery recycling

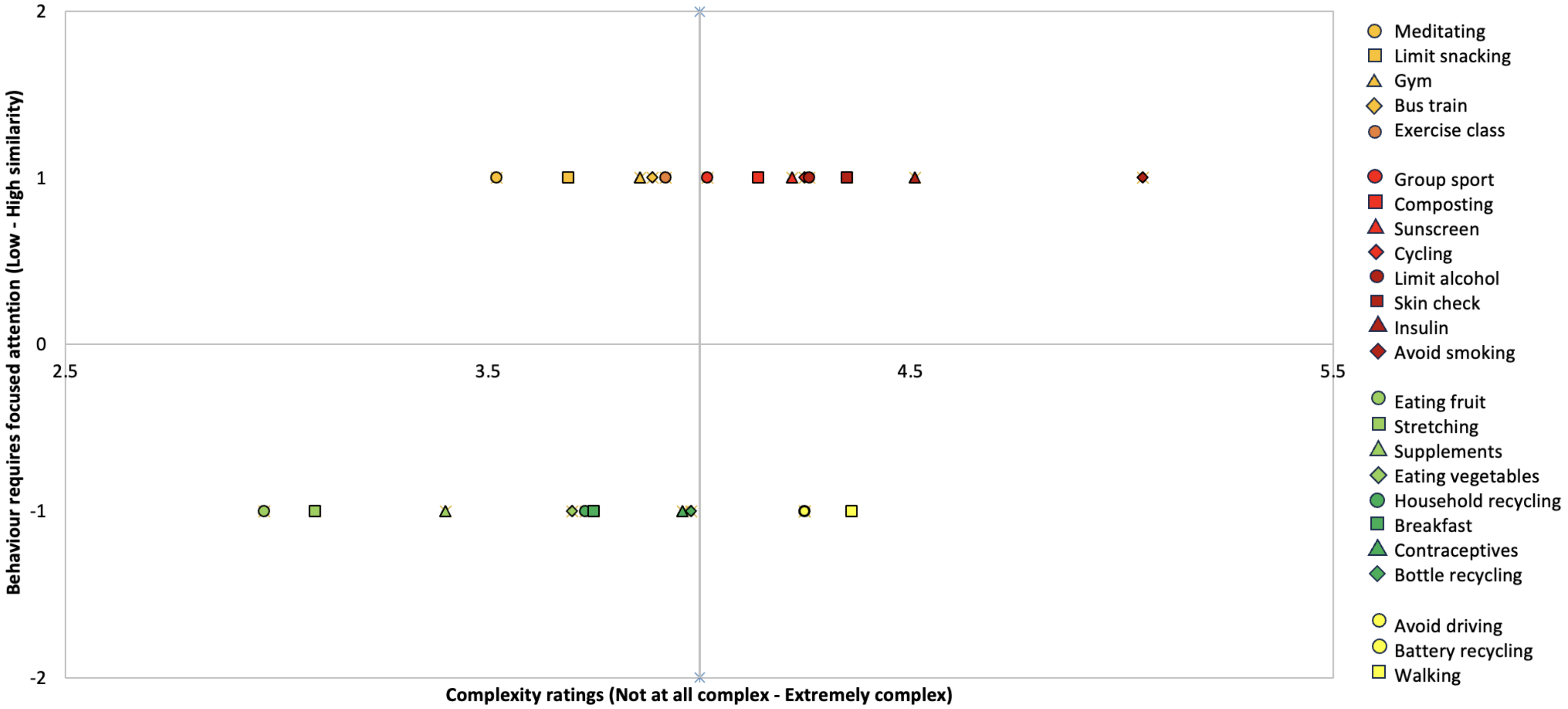
2
1
0
-1
-2
2.5
3.5
4.5
5.5
Behaviour requires focused attention (Low - High similarity)
Complexity ratings (Not at all complex - Extremely complex)
Meditating
Limit snacking
Gym
Bus train
Exercise class
Group sport
Composting
Sunscreen
Cycling
Limit alcohol
Skin check
Insulin
Avoid smoking
Eating fruit
Stretching
Supplements
Eating vegetables
Household recycling
Breakfast
Contraceptives
Bottle recycling
Avoid driving
Battery recycling
Walking

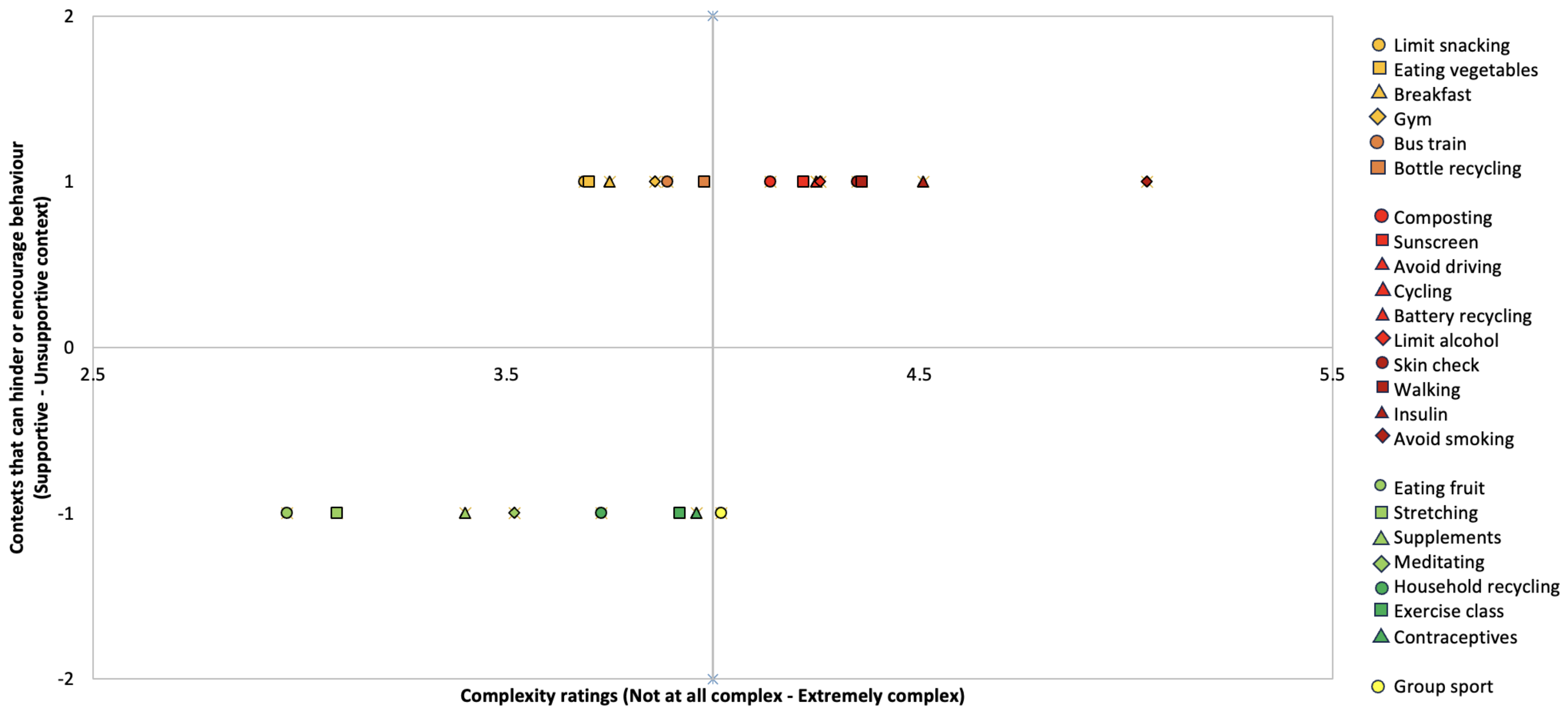
Contexts that can hinder or encourage behaviour
(Supportive - Unsupportive context)
2
1
0
-1
-2
2.5
3.5
4.5
5.5
Complexity ratings (Not at all complex - Extremely complex)
Limit snacking
Eating vegetables
Breakfast
Gym
Bus train
Bottle recycling
Composting
Sunscreen
Avoid driving
Cycling
Battery recycling
Limit alcohol
Skin check
Walking
Insulin
Avoid smoking
Eating fruit
Stretching
Supplements
Meditating
Household recycling
Exercise class
Contraceptives
Group sport

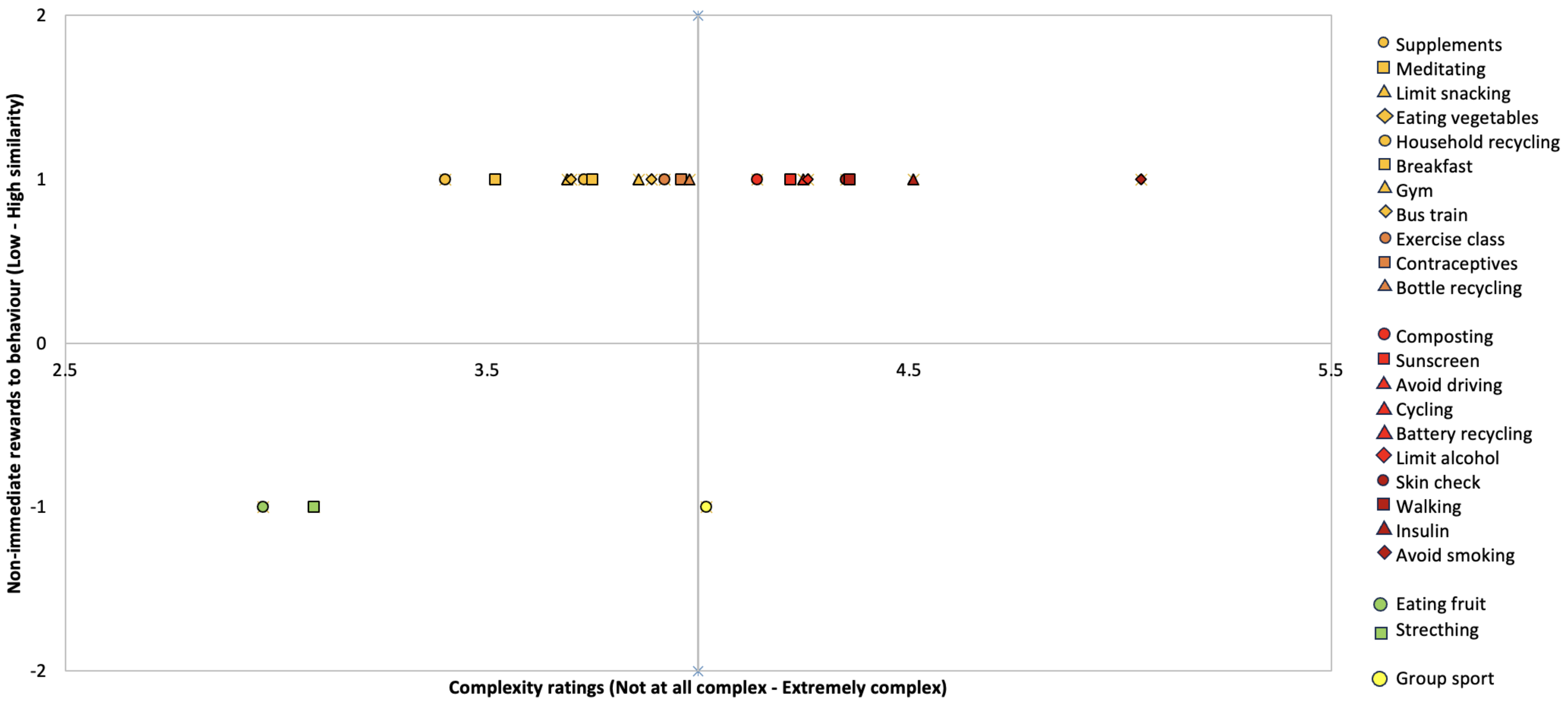
2
1
0
-1
-2
2.5
3.5
4.5
5.5
Non-immediate rewards to behaviour (Low - High similarity)
Complexity ratings (Not at all complex - Extremely complex)
Supplements
Meditating
Limit snacking
Eating vegetables
Household recycling
Breakfast
Gym
Bus train
Exercise class
Contraceptives
Bottle recycling
Composting
Sunscreen
Avoid driving
Cycling
Battery recycling
Limit alcohol
Skin check
Walking
Insulin
Avoid smoking
Eating fruit
Strecthing
Group sport

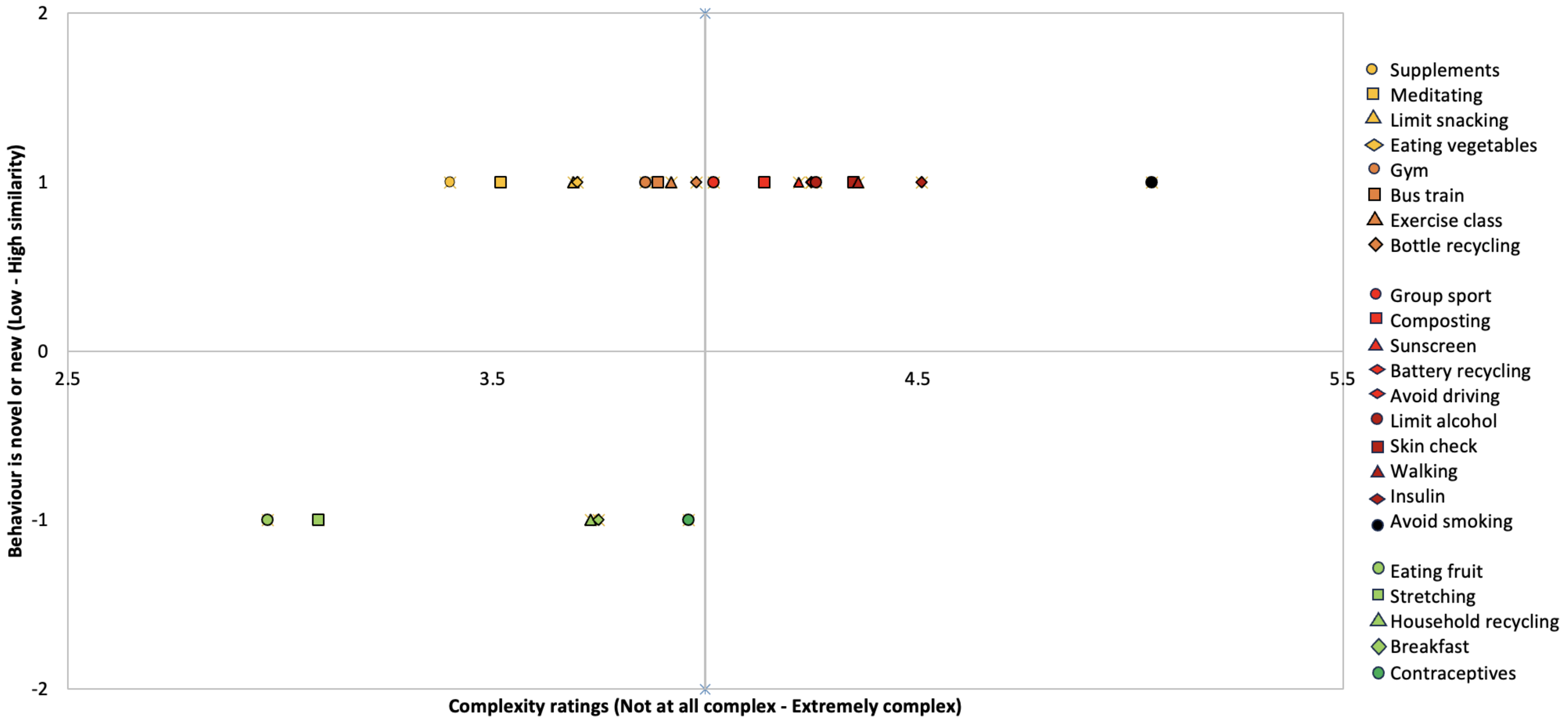

Note. In each figure, behaviors were plotted based on the mean degrees of complexity.

Behaviors indicated with the same shape and colour were rated the same mean degrees of complexity.
